# MicroRNA-21 induces loss of 15-hydroxyprostaglandin dehydrogenase in early gastric tubular adenocarcinoma

**DOI:** 10.1038/s41598-018-36139-z

**Published:** 2018-12-07

**Authors:** Young Soo Park, Jeong Hoon Lee, Deok-Beom Jung, Han-Byul Kim, Jin-Hak Jung, Sehyung Pak, Yeon-Mi Ryu, Hye Jin Park, Yun-Yong Park, Hwoon-Yong Jung, Seung-Jae Myung

**Affiliations:** 1 Departments of Pathology, University of Ulsan College of Medicine, Asan Medical Center, Seoul, Korea; 2 Gastroenterology, University of Ulsan College of Medicine, Asan Medical Center, Seoul, Korea; 3 Biomedical Research Center, Asan Institute for Life Sciences, Seoul, Korea

## Abstract

15-hydroxyprostaglandin dehydrogenase (15-PGDH), the rate-limiting enzyme in prostaglandin E2 degradation, is decreased in gastric cancers and microRNA (miR)-21 is one of the regulators. We investigated the expression and regulation of 15-PGDH in eary gastric carcinogenesis utilizing endoscopic submucosal dissection (ESD) and gastric cancer cell lines. Expression of 15-PGDH and cyclooxygenase-2 as well as the promoter methylation of 15-PGDH were evaluted. CRISPR, miR-21 transfection, proliferation and apoptosis assays were also done. We observed significant decreases in 15-PGDH expression but no promoter methylation was detected in any ESDs. 15-PGDH suppression by CRISPR induced enhanced growth kinetics. miR-21, which was detected in high level in gastric tumors from the TGCA data, caused increased proliferation, decreased apoptosis. miR-21 overexpression was confirmed with CISH and RT-PCR in the ESDs. Loss of 15-PGDH occurs at the very early stage of gastric adenocarcinoma by miR-21. *H*. *pylori* infection may affect miR-21 up regulation. Maintaining 15-PGDH enzyme activity could be a new strategic measure in preventing gastric cancer especially tubular adenocarcinoma.

## Introduction

The degradation of prostaglandins (PG) by 15-hydroxyprostaglandin dehydrogenase (15-PGDH) is one of the crucial steps in regulating PG levels, especially prostaglandin E2 (PGE_2_), which is known to play major roles in carcinogenesis, cancer progression, and tissue regeneration^1–3^. PGE_2_ is produced from arachidonic acid by cyclooxygenase-1 (COX-1) or COX-2 followed by PGE_2_ synthase^4^, and then it is degraded by 15-PGDH. Overexpression of 15-PGDH therefore leads to a reduction in PGE_2_ level, acting as an antagonistic enzyme to COX-2. Moreover, 15-PGDH has been shown to act as a tumor suppressor in gastrointestinal cancers, such as colon cancers^5–7^.

Previous studies suggest that 15-PGDH is associated with gastric cancers as well. Although its incidence is gradually decreasing, gastric cancer is the fourth leading cancer and second most common cause of cancer-related deaths worldwide, especially in Eastern Asia^8^. Our previous studies demonstrated loss of 15-PGDH expression in gastric cancers and adenomas^9^ and showed that 15-PGDH was inhibited by *Helicobacter pylori* (*H*. *pylori*) infection^10^. Other studies also reported decreased expression of 15-PGDH in gastric cancers^11–13^, and its role as a poor prognostic factor^14,15^. However, these studies used tumor specimens from surgical resection, and a large proportion was from patients with advanced gastric cancer. Because the function 15-PGDH as a tumor suppressor has been studied in early carcinogenesis, such as in colonic adenomas and aberrant crypt foci^5^, we targeted early tubular adenocarcinomas with tubular adenoma in the background. Because our goal was to unravel the role of 15-PGDH in the earliest gastric carcinogenesis, we collected specimens from endoscopic submucosal dissection (ESD), which is an established treatment for early gastric cancer^16^.

In addition, we attempted to elucidate how 15-PGDH was regulated in early carcinogenesis. A few possible inhibitory mechanisms were reported previously, including promoter methylation in gastric cancers^12^ and microRNA(miRNA) in cholangiocarcinomas^17^. In this study, methylation status was evaluated using methylation-specific polymerase chain reaction (MS-PCR), as well as pyrosequencing. Using the public data from The Cancer Genome Atlas (TCGA)^18^, we found a negative correlation between microRNA-21 (miR-21) and 15-PDGH. Along with assessing the expression profile of miR-21, functional analysis was conducted after transfecting miR-21 to gastric cancer cell lines.

Herein, we investigated the expression pattern and regulation of 15-PGDH using gastric cancer cell lines, as well as gastric ESD specimens.

## Results

### 15-PGDH expression was significantly decreased in early gastric tubular adenocarcinoma

**Figure 1:**
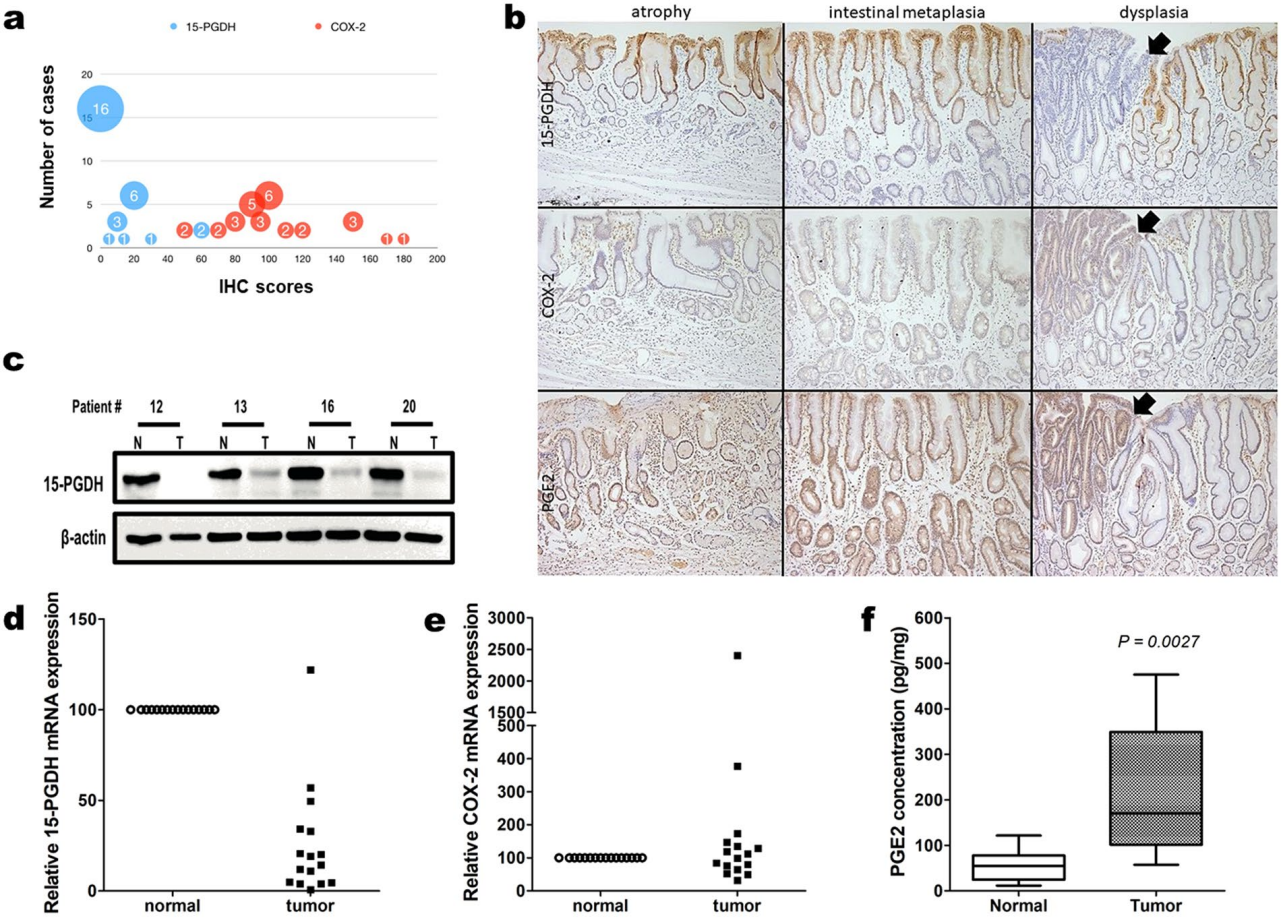
(**a**) IHC scores of 15-PDGH (blue) and COX-2 (orange) expression in tumors from 30 cases of retrospectively collected ESD specimens. The paired t-test revealed a significant difference. (**b**) representative photomicrographs of 15-PGDH, COX-2, and PGE2 IHC in atrophy, intestinal metaplasia, and dysplasia (original magnification, x100). 15-PGDH (top row) lost its expression at dysplasia (arrows), but was visible until intestinal metaplasia. COX-2 (middle row) was weakly positive in intestinal metaplasia and strongly positive in dysplasia. PGE2 (bottom row) exhibited its highest expression in the dysplastic focus. (**c**) representative 15-PGDH WB from prospectively collected patient samples. Expression was markedly decreased or absent in tumor (T) samples relative to normal samples (N). (**d**,**e**) relative expression of 15-PGDH (*HPGD*, **d**) and COX-2 (*PTGS2*, **e**) mRNA in tumor samples relative to normal samples from patients. 15-PGDH (*HPGD*, **d**) exhibited significantly lower levels in the tumor samples. Some of the samples showed high COX-2 (*PTGS2*, **e**) levels, but it was not statistically significant. (**f**), the PGE2 concentration level was higher in the tumor samples. Error bars denote 5 to 95 percentile.

The expression of 15-PGDH, COX-2, and PGE_2_ was evaluated via IHC using FFPE sections from 30 ESD specimens. Because these retrospectively collected specimens were very early lesions with tubular adenoma in the background, normal/atrophic and intestinal metaplastic mucosa were always present in the vicinity. The carcinoma and tubular adenoma area of these specimens consistently exhibited very low or absence of 15-PGDH, whereas COX-2 was intermediately or strongly expressed (Fig. 1a). The IHC staining scores of 15-PGDH were significantly lower than that of COX-2 (paired t-test, *P* < 0.0001).

We then compared the expression patterns of these proteins in adjacent normal/atrophic or intestinal metaplastic mucosa (Fig. 1b). 15-PGDH, which was well expressed in normal/atrophic and metaplastic foveolar epithelial cells, disappeared in dysplastic cells (tubular adenoma and/or adenocarcinoma). COX-2, on the other hand, was not expressed in normal/atrophic epithelial cells, but started to show positivity in metaplastic cells and revealed higher expression in dysplastic cells. PGE_2_ exhibited a similar pattern as that of COX-2. The loss of 15-PGDH and high COX-2 and PGE_2_ persisted in the carcinoma area (Supplementary Fig. 1).

This was further confirmed with snap frozen tumor/normal samples collected from 16 patients. The samples were collected with endoscopic forceps before performing ESD. All were confirmed to have early tubular adenocarcinomas in FFPE sections. 15-PGDH WB was lost or exhibited significantly decreased expression in all tumor tissues compared to the normal samples (Fig. 1c). Decreased (hydroxyprostaglandin dehydrogenase 15 (15-PGDH, *HPGD*) mRNA expression in tumor samples relative to that of normal samples was obvious (Fig. 1d), whereas prostaglandin-endoperoxide synthase 2 (COX-2, *PTGS2*) mRNA failed to present a difference between tumor and normal samples (Fig. 1e). On the other hand, the PGE_2_ level was increased in the tumor samples (Fig. 1f).

### 15-PGDH and COX-2 expression pattern in gastric cancer cell lines

**Figure 2:**
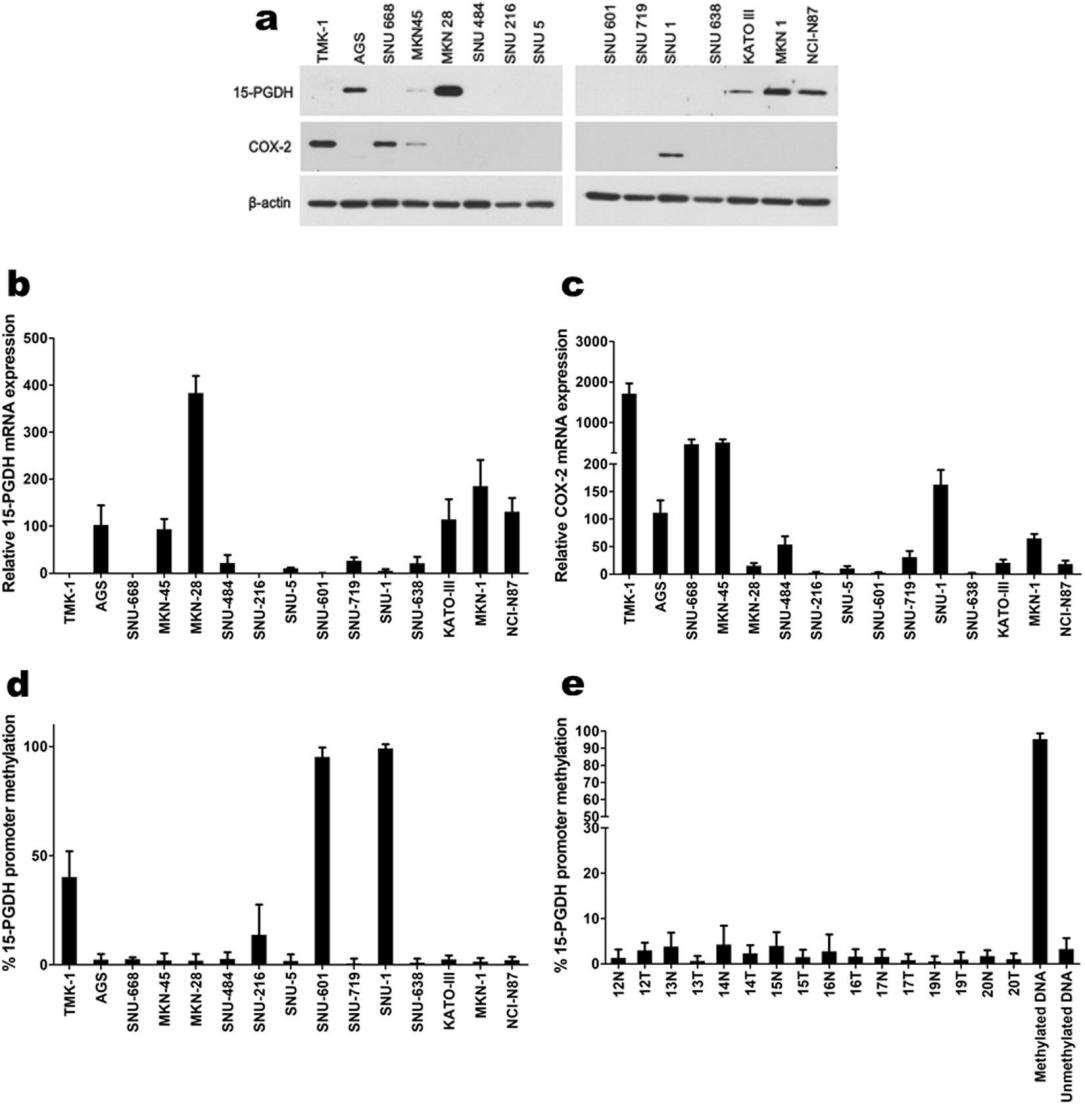
(**a**) WB expression pattern of 15-PGDH and COX-2 in gastric cancer cell lines. (**b**,**c**), relative 15-PGDH (*HPGD*, **b**) and COX-2 (*PTGS2*, **c**) mRNA expression profiles in gastric cancer cell lines (expression of AGS was used as the standard = 100). The protein expression and mRNA expression exhibited strong correlations. The results are summarized in Supplementary Table 1. (**d**,**e**), 15-PGDH methylation assay by pyrosequencing in gastric cancer cell lines (**d**) and patient samples (**e**). Some of the cancer cells with no 15-PGDH expression showed methylation (TMK-1, SNU-216, SNU-601, SNU-1), whereas others did not (SNU-484, SNU-5, SNU-719, SNU-638). Error bars denote standard deviations.

The protein and mRNA expression of 15-PGDH and COX-2 was screened using various gastric cancer cell lines. Protein expression by WB (Fig. 2a) showed good correlation with mRNA expression by qRT-PCR (Fig. 2b,c). Based on these results, we could categorize gastric cancer cell lines in terms of 15-PGDH and COX-2 expression (Supplementary Table 1). TMK-1, SNU-668, and SNU-1 exhibited loss of 15-PGDH and high COX-2 expression. AGS, MKN-28, KATO III, MKN-1, and NCI-N87 had high 15-PGDH and no COX-2 expression. The MKN-45 cell line expressed both 15-PGDH and COX-2. The rest of the cells lines (SNU-484, SNU-216, SNU-5, SNU-601, SNU-719, and SNU-638) expressed neither 15-PGDH nor COX-2.

### Methylation status does not correlate 15-PGDH expression

Because the 15-PGDH promoter region methylation was reported to be one of the mechanisms of regulating 15-PGDH, we measured methylation by pyrosequencing in previously screened gastric cancer cells lines. The cells that had methylation (TMK-1, SNU-216, SNU-601, and SNU-1) did not express 15-PGDH in WB. However, other cells (such as SNU-484, SNU-5, SNU-719, and SNU-638) bared no methylation even though no 15-PGDH was expressed (Supplementary Table 1 and Fig. 2d). This discrepancy with 15-PGDH expression and methylation was seen in patient samples as well. None of the patient samples had enough methylation to account for the loss of 15-PGDH protein expression (Fig. 2e). This was also confirmed with MS-PCR (data not shown). Two cells lines (TMK-1 and SNU-601) were selected to determine if the de-methylation of 15-PGDH with 5-aza-dC treatment induced protein expression. Up to 50 µM of 5-aza-dC was used, but it had little or no effect on the promoter site de-methylation or protein expression (Supplementary Fig. 2ab), although DNMT1 expression was decreased.

### 15-PGDH suppression enhances proliferation of gastric cancer cell lines

**Figure 3:**
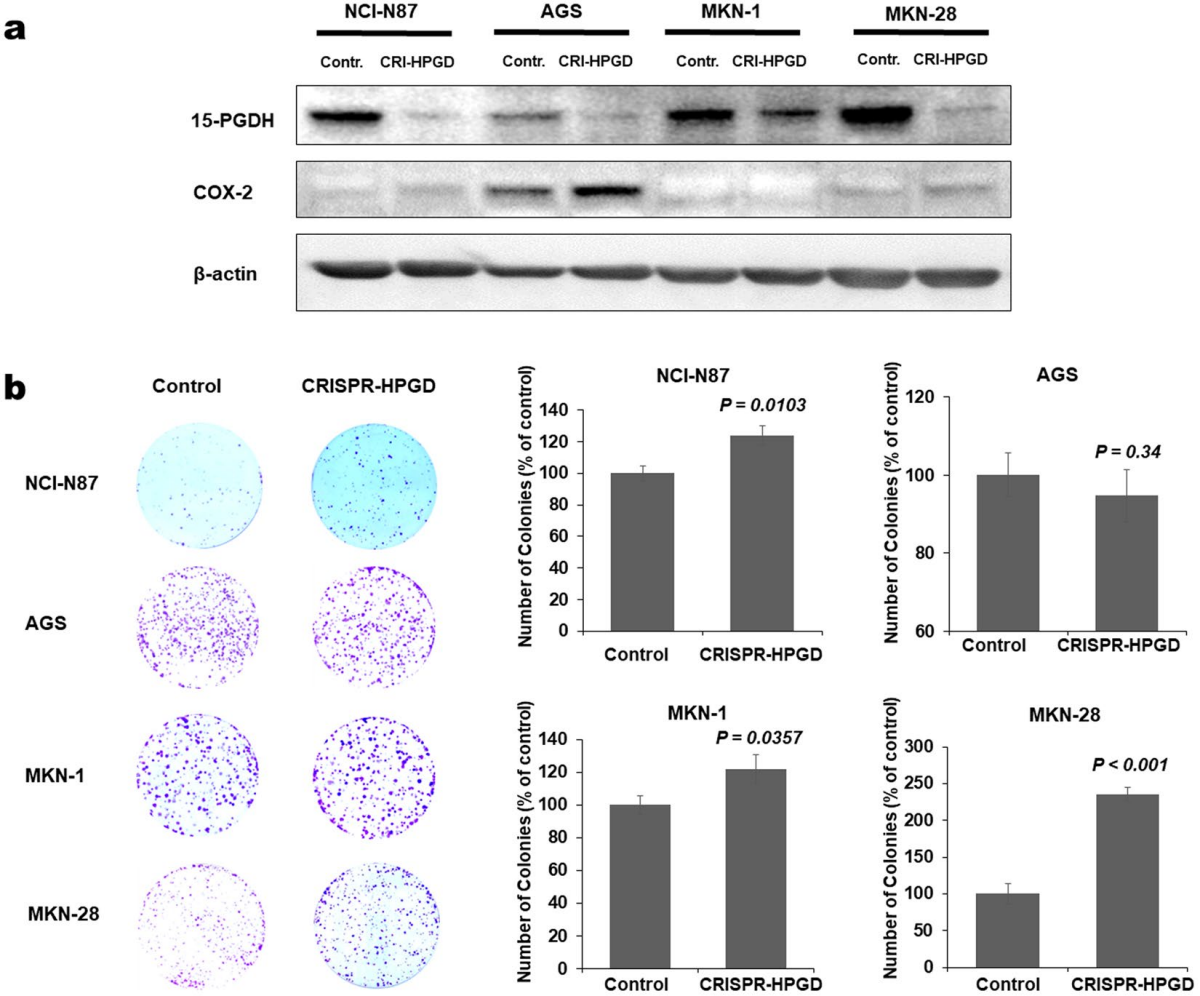
15-PGDH suppression using CRISPR-Cas9 system in gastric cancer cell lines. (**a**) WB assay confirmed 15-PGDH suppression. (**b)** colony forming assay after 15-PGDH (*HPGD*) suppression showed increased number of colony forming units in NCI-N87, MKN-1 and MKN-28.

Although we were not able to reverse methylation status to induce 15-PGDH expression, we instead suppressed 15-PGDH expression using the CRISPR-Cas9 system. We chose gastric cancer cell lines that expressed 15-PGDH (AGS, MKN-28, MKN-1, and NCI-N87) and suppressed 15-PDGH expressions but COX-2 levels were unaltered (Fig. 3a). The numbers of colonies were significantly increased in MKN-28, MKN-1, and NCI-N87 after 15-PGDH (*HPGD*) suppression (Fig. 3b). On the other hand, overexpressing 15-PDGH in cell lines with low 15-PGDH (KATO III, MKN 45 and SNU 1) inhibited the growth kinetics (Supplementary Fig. 3).

### miR-21 is upregulated in gastric cancers

**Figure 4:**
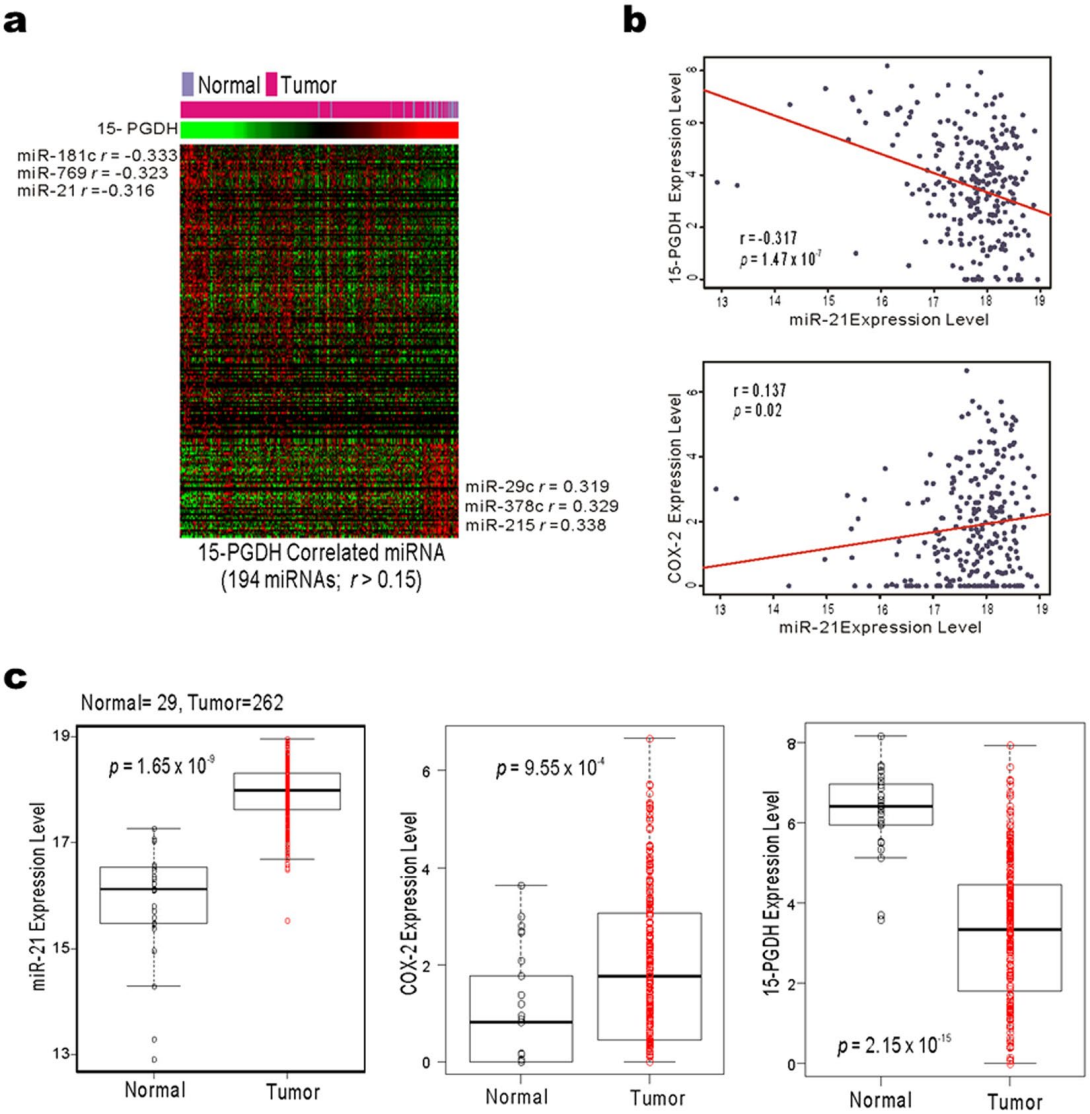
Correlation of 15-PGDH, COX-2, and miR-21 from TCGA gastric cancer data analysis. (**a**) TCGA gastric cancer data, miR-181, miR-769, and miR-21 showed high correlation with 15-PGDH (*HPGD*). (**b**) the miR-21 expression level was negatively correlated with 15-PGDH (*HPGD*) and positively correlated with COX-2 (*PTGS2*). (**c**) the levels of miR-21 and COX-2 (*PTGS2*) were higher in the tumor tissue than in normal tissue, but 15-PGDH (*HPGD*) mRNA expression was lower in the tumor tissue.

In search for other possible mechanisms for 15-PGDH expression regulation, we determined the correlation of 194 miRNAs with 15-PGDH from the TCGA gastric cancer public database^18^. Three miRNAs (miR-181c, miR-769, and miR-21) were reversely correlated with the 15-PGDH (*HPGD*) gene (Fig. 4a). We considered miR-21 a good candidate for further study because miR-21 has been reported to have a binding site in 3′-UTR of 15-PGDH mRNA^17^.

A negative relationship was found between miR-21 and 15-PGDH (*HPGD*) mRNA expression levels (Fig. 4b, r = −0.317, *P* < 0.001) and there was a positive correlation between miR-21 and COX-2 (*PTGS2*) mRNA expression (Fig. 4b, r = 0.137, *P* = 0.02). miR-21 and COX-2 (*PTGS2*) mRNA levels were higher in the tumor samples than normal tissue, whereas 15-PGDH (*HPGD*) mRNA was significantly lower in the tumor samples of the TCGA cohort (Fig. 4c).

### miR-21 downregulates 15-PGDH expression

**Figure 5:**
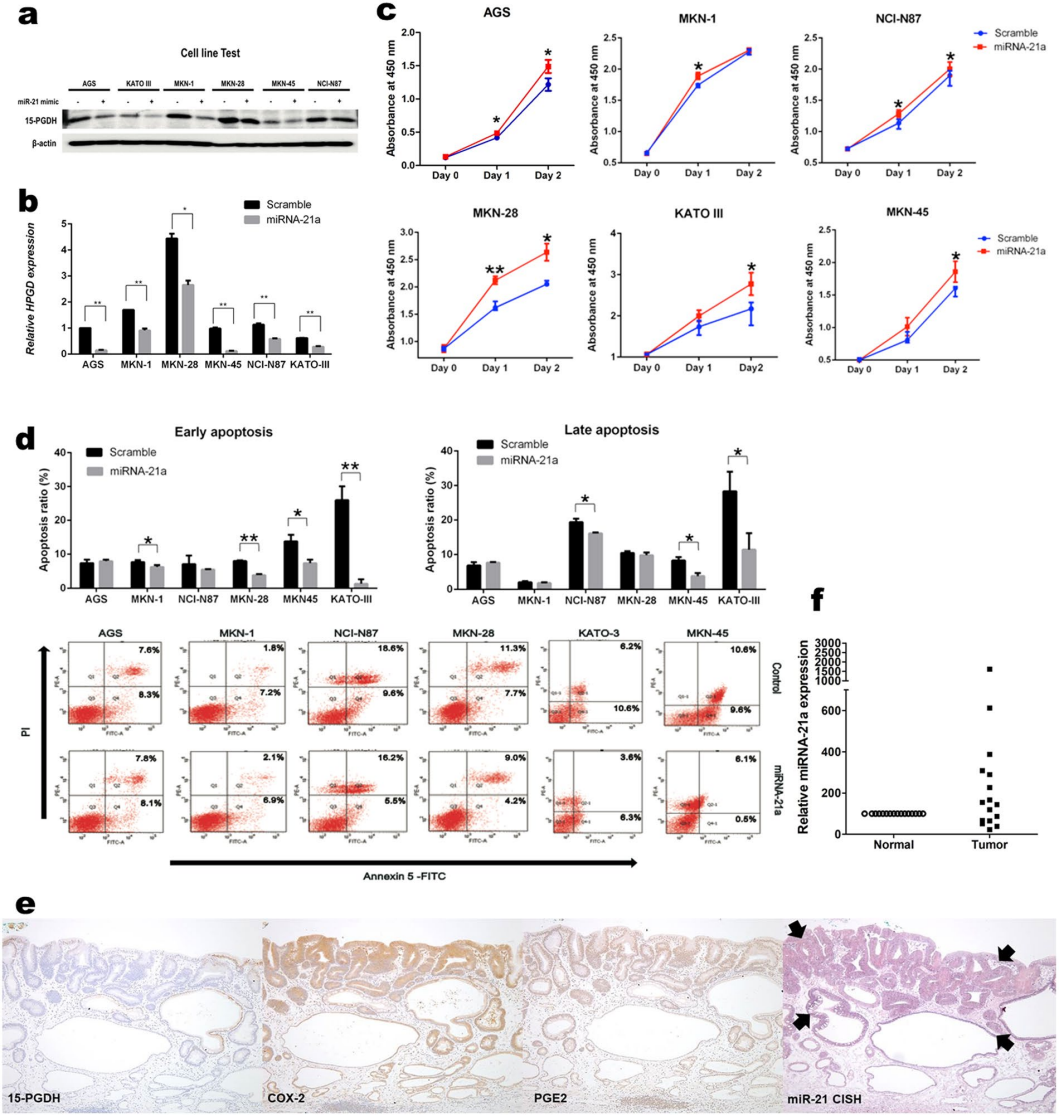
(**a**,**b**) 15-PGDH expression change after miR-21 transfection to gastric cancer cell lines. Protein expression (**a**), as well as mRNA expression (**b**), was inhibited after transfection. (**c**,**d**) proliferation (**c**) and apoptosis (**d**) assay of gastric cancer cell lines after miR-21 transfection. Cells showed increased proliferation and decreased apoptosis when miR-21 was transfected. Data are mean values from three independent experiments, (**b**–**d**, *P < 0.05, **P < 0.001). (**e**) representative photomicrographs of miR-21 CISH from patient samples. In the tumor area (arrows), 15-PGDH was not expressed, whereas COX-2, PGE2, and miR-21 were high. (**d**) the relative expression of miR-21 was also higher in the patient tumor samples.

We chose six gastric cancer cell lines that expressed 15-PGDH in WB (AGS, KATO III, MKN-1, MKN-28, MKN-45, and NCI-N87) to functionally validate the correlation of miR-21 and 15-PGDH expression. When miR-21 was transfected, all six cell lines showed diminished 15-PGDH protein expression (Fig. 5a). The mRNA expression alteration after miR-21 transfection correlated well with the protein, which was confirmed by qRT-PCR (Fig. 5b).

The effect miR-21 on cell proliferation and apoptosis was further studied using these six cell lines. The proliferation assay showed a significant increase in cell growth after 2 days of transfection in AGS, MKN-28, KATO III, and MKN-45 (Fig. 5c). In the apoptosis assay, KATO III and MKN-45 showed decreased early and late apoptosis (Fig. 5d). Colony formation assay using miR-21 transfected cells also confirmed increase cell growth (Supplementary Fig. 4).

miR-21 CISH in patient tissue also confirmed that miR-21 was increased in tumor areas (Fig. 5e). The CISH score was higher in the tumor area than the adjacent normal area (2.3 ± 0.2 vs. 0.75 ± 0.2, paired t-test, *P* < 0.0001) and relative miR-21 expression measured by qRT-PCR was significantly higher in the tumor area (*P* = 0.03, Fig. 5f). We performed miR-21 CISH on another patient set (retrospectively collected 30 cases of ESD). The CISH score was also higher in the tumor area (1.87 ± 1.07 vs. 0.33 ± 0.48, *P* < 0.0001, Supplementary Fig. 5).

### miR-21 was increased in patients with *H*. *pylori* infection

Finally, we compared miR-21 expression with *H*. *pylori* infection status. Applying CISH, we were able to show that miR-21 was increased in *H*. *pylori*-infected patients in biopsies at both the antrum and body (Supplementary Fig. 6A,B). This was done using the cohort from the previous study where we demonstrated that 15-PGDH expression was inhibited by *H*. *pylori* infection^10^. When *H*. *pylori* was infected to AGS, miR-21 mRNA expression increased (*P* = 0.008, Supplementary Fig. 6C). Subgroup analysis with 16 ESD patients (13 *H*. *pylori* infected subjects) revealed that miR-21 CISH scores were higher in tumor tissues compared to normal tissues from *H*. *pylori*-infected patients, but not in patients with no *H*. *pylori* infection (*P* < 0.0001, Supplementary Fig. 6D). In addition, miR-21 was higher in the tumor tissues of *H*. *pylori*-infected patients than the tumor tissues of uninfected patients (*P* = 0.012, Supplementary Fig. 6D).

## Discussion

In this study, we demonstrated that 15-PGDH expression is lost during early carcinogenesis of gastric cancer. Unlike diffuse-type gastric carcinomas, tubular adenocarcinomas or intestinal-type gastric carcinomas are believed to undergo an adenoma-carcinoma sequence similar to colorectal cancers, although specific alteration of genes involved in the process are quite different^19,20^. Loss of 15-PGDH promotes the earliest steps of colon carcinogenesis^21^ and we observed this phenomenon in the early gastric carcinogenesis as well, using the ESD specimens. The expression of 15-PGDH was maintained until atrophy or intestinal metaplasia, but disappeared in dysplasia (from adenoma to carcinoma). On the other hand, COX-2 expression started to increase from metaplasia throughout dysplasia. Loss of 15-PGDH combined with COX-2 increase would result in PGE_2_ accumulation and this was partly verified either by IHC or mass spectrometry. This observation advocates the role of 15-PGDH as a gatekeeping tumor suppressor. On the contrary, we observed persistent 15-PGDH expression in early signet ring cell carcinoma (Supplementary Fig. 7), especially when the tumor was confined within the lamina propria. Signet ring cell carcinoma is a typical diffuse-type gastric carcinoma and it is believed to undergo a different pathway of carcinogenesis.

One of the proposed mechanism of 15-PGDH down regulation in the literature is the promoter methylation. This was observed not only in advanced gastric cancer^12^, but in non-neoplastic gastric mucosa with *H*. *pylori* infection as well^10^. However, our results were to the contrary. No methylation was detected in all of the patient samples and in some of the gastric cancer cell lines with no 15-PGDH expression. Jang *et al*. also said that they did not observe promoter methylation in advanced gastric cancers^13^. It may be possible that methylation was responsible for 15-PGDH loss in a certain subset of patients, but miR-21 seemed more involved in 15-PGDH inhibition in early carcinogenesis according to our data. miR is a small non-coding RNA molecule, that functions in RNA silencing and post-transcriptional regulation of gene expression. In previous studies, Lu *et al*.^17^ and Li *et al*.^22^ found miR-21 binding sites in the 3′UTR of 15-PGDH mRNA using microRNA.org resource. In the present study, we showed that transfected miR-21 inhibited 15-PGDH, which in turn enhanced cell proliferation and reduced apoptosis. 15-PGDH suppression by CRISPR-Cas9 also increased proliferation, which is in agreement with the results from Li *et al*. who saw inhibition of cell proliferation by transfecting 15-PGDH^23^. However, miR-21 is a well-known oncomiR and overexpressed in many solid tumors. In addition, 15-PGDH is not the only possible target that miR-21 inhibits. Many studies have advocated that miR-21 enhances gastric cancer growth though PTEN^24,25^ and this regulates sensitivity to certain drugs^26,27^. miR-21 has also been studied in other gastrointestinal cancers, such as cholangiocarcinoma^17^, and colorectal cancer^28,29^. Therefore, it is crucial to provide additional evidence that miR-21 binds to 15-PGDH mRNA by immunoprecipitation methods, such as RNA immunoprecipitation or crosslinking and immunoprecipitation^30^. In addition, it would be interesting to determine if PTEN is involved in 15-PGDH inhibition as well. Other possible mechanisms responsible for 15-PGDH loss would be COX-2. Liu *et al*. demonstrated that COX-2 down regulates 15-PGDH^31^. We also observed an inverse correlation with COX-2 and 15-PGDH in some gastric cancer cell lines, as well as in a few patient samples, but there were also cancer cell lines and cases that did not express COX-2 even at the absence of 15-PGDH, suggesting other regulatory mechanisms are in play.

*H*. *pylori* is considered a Group I carcinogen by the International Agency for Research on Cancer^32^ and it is known to cause genetic and epigenetic changes^33^, as well as PGE_2_ synthesis^34^. We have previously shown that *H*. *pylori* infection inhibits 15-PGDH expression and this was associated with epidermal growth factor receptor (EGFR) and Snail^10^. Zhao *et al*. also reported that *H*. *pylori* infection was strongly associated with 15-PGDH loss^35^. The data from the current study suggested that miR-21 might be also be involved in early carcinogenesis because of persistent *H*. *pylori* infection. However, this question was far beyond the scope of the present study because much more in-depth investigation is needed to unravel a direct correlation of *H*. *pylori* infection and miR-21.

In summary, we have shown that 15-PGDH is markedly lower during early carcinogenesis of gastric tubular adenocarcinoma and is regulated by miR-21, but not by methylation. We have also observed that *H*. *pylori* infection is correlated with an increase in miR-21, which requires further research. Maintaining 15-PGDH enzyme activity would be a new strategic measure in preventing tubular adenocarcinoma of the stomach.

## Materials and Methods

### Patients and tissue specimens

Three groups of human samples were used in this study. First, 30 ESD cases of well-differentiated adenocarcinomas were retrospectively collected between 2007 and 2008. All were very early lesions harboring tubular adenoma (low or high grade) and intestinal metaplasia in the background. Second, 20 more ESD cases were prospectively enrolled in 2014. Well-differentiated adenocarcinomas eligible for ESD were targeted and four forcep biopsies were taken from tumor and normal areas. Top frozen section slides were histologically evaluated for the proportion of tumor tissue. Four cases were excluded because of the lack of tumor tissue and 16 cases were used in this study. Lastly, we used formalin-fixed paraffin-embedded (FFPE) tissues from 26 patients enrolled in our previous study^10^. All patients had paired biopsy from the body and antrum. All studies using human specimens adhered to the guidelines established by the Declaration of Helsinki, and were approved by the institutional review board of the Asan Medical Center. We obtained informed consent from all human participants.

### Gastric cancer cell lines

Fifteen gastric cancer cell lines were used in this study (TMK-1, AGS, KATO III, NCI-N87, MKN-1, MKN-28, MKN-45, SNU-1, SNU-5, SNU-216, SNU-484, SNU-601, SNU-638, SNU-668, and SNU-719). Gastric cancer cell lines were maintained in RPMI 1640 medium containing 10% fetal bovine serum (Gibco), 100 U/mL penicillin, and 100 μg/mL streptomycin in a 5% CO_2_ atmosphere.

### Immunohistochemical (IHC) staining

Five-micrometer sections were deparaffinized and gradually rehydrated. Antigen retrieval was accomplished by heating the slides at 96 °C for 60 min in 10 mM citrate buffer (pH 6.0), followed by cooling for 20 min. Endogenous peroxidase activity was blocked by soaking of sections in hydrogen peroxidase (S2023; DAKO) for 5 min. Avidin-biotin blocking was performed for 5 min, followed by nonspecific protein blocking (X0909; Serum-Free Protein Block; Dako). Sections were washed in Tris-buffered saline (TBS; 50 mM Tris·HCl/150 mM NaCl; pH 7.6) for 10 min and incubated overnight with the primary antibodies (supplementary materials). Slides were washed with TBS, incubated with the Dako Envision System Kit solution (K5007; Dako) for 30 min, developed with diaminobenzidine solution (Dako), washed with distilled water, and counterstained with hematoxylin.

### Western blotting (WB)

For protein extraction, cells were lysed by RIPA buffer (Thermo) with a cocktail of protease and phosphatase. Protein lysates were separated on SDS-polyacrylamide gels and then transferred to PVDF membranes (Bio-Rad). Membranes were blocked in TBST buffer containing 5% bovine serum albumin (BSA) for 1 h at room temperature (RT) and probed with primary antibodies (supplementary materials) overnight. The membrane was then incubated in horseradish peroxidase-linked secondary antibodies (cell signaling) diluted 1:5000 in 5% BSA. Blots were developed using a Supersignal West pico chemiluminescent kit (Thermo).

### Quantitative reverse transcription polymerase chain reaction (qRT-PCR) analysis

qRT-PCR was performed with Applied Biosystem® 7900 Real-time PCR systems. For the 15-PGDH, COX-2, and GAPDH assays, total RNA was extracted using RNeasy mini Kit (Qiagen). RNA was reverse transcribed using MultiScribe^TM^ Reverse Transcriptase (Applied Biosystems). For the miR-21 assay, RNA was isolated using miRNeasy (Qiagen). cDNA was synthesized using the miScript II RT kit (Qiagen) according to the manufacturer’s instructions. miRNA was detected using the miScript SYBR® Green PCR kit (Qiagen). Primer information is in the supplementary materials. The expression results were analyzed using SDS 2.2.1 software (Applied Biosystems). The gene of interest was normalized to GAPDH results. DATA were determined by the comparative 2^−△Ct^ method.

### PGE_2_ quantification

PGE_2_ was extracted from frozen samples of human or media of gastric cancer cells and analyzed by reverse-phase liquid chromatography mass spectrometry^5^.

### Methylation analysis using MS-PCR and pyrosequencing

Genomic DNA of gastric cancer cell lines was isolated with a DNeasy blood & tissue kit (Qiagen). Bisulfite modification was performed by the EZ DNA methylation-Gold kit (Zymo Research). The primer pairs were designed to detect un-methylated (U) or methylated (M) bisulfite converted 15-PGDH promoter. MS-PCR was performed using the EpiTect MSP Kit (Qiagen). Pyrosequencing analysis was performed using each primer designed by the PSQ assay design program (Biotage). The percentage of 15-PGDH methylation was determined by calculating the average methylation at 10 CpG sites in pyrosequencing. Primer sequences and reaction details are described in the supplementary materials.

### 5-Aza-2′-deoxycytidine (5-aza-dC) treatment

Cells were incubated for 72 h with 0, 10, 25, and 50 mM 5-aza-dc (Sigma Chemical Co., St. Louis, MO) with the culture media being replaced every 24 h with fresh media containing 5-aza-dC. After incubation for 72 h, the cells were harvested for protein isolation.

### Genomic data from the TCGA cohort

Genomic data from the TCGA gastric cancer cohort were downloaded from the TCGA data portal site (http://cancergenome.nih.gov/) and processed as described in previous studies^18^. We analyzed mRNA matched miRNA sequencing data.

### miRNA assay using qRT-PCR, chromogenic *in situ* hybridization (CISH), and transfection

qRT-PCR was performed for miR-21 as described above. CISH was performed on FFPE tissue sections using a miRCURY LNA microRNA detection kit (Exiqon, details in supplementary materials). For the miR-21 transfection study, 15-PGDH-directed miR-21 (UAGUCAGACUAUUCGAu) was constructed as previously described^17^. The miR-21 and scramble mimic were transfected into gastric cancer cells using RNAiMAX (Invitrogen) according to the manufacturer’s instructions.

### Proliferation and apoptosis assay

miR-21 transfected cell lines were seeded at a density of 1 × 10^4^ cells/well in 96-well plates using an automated cell counter (Luna). Cell proliferation assays were performed according to the manufacturer’s instructions (WST-1 reagent, ROCHE). The absorbance of solution was measured according to the manufacturer’s protocol using an ELISA reader.

The apoptosis assay was analyzed by flow cytometry, which measures cells positively stained using the Annexin V-FITC apoptosis detection kit (BD Pharmingen) according to the manufacturer’s instructions. Flow cytometry was performed on BD FACSCanto™ (BD Biosciences). Data from a total of 10,000 events were analyzed using BD FACSCanto™ clinical software v2.4 (BD Biosciences).

### Colony forming assay using the CRISPR-Cas9 system

Guide RNA (gRNA) was designed using the web-tool CHOP-CHOP (http://chopchop.cbu.uib.no/). gRNA for 15-PGDH knock-out primer was: gRNA1 sense, 5-CAC CGT TGT CTA TAG GTA GCG CTG G-3; anti-sense, 5-AAA CCC AGC GCT ACC TAT AGA CAA C-3. gRNA cloned lentiCRISPRv2.0 was restricted by BsmB (New England Biolabs). Knock-out viral vectors (Viral vector: PLP1: PLP2: VSV-G) were mixed at a 9:6:6:3 (μg) ratio with 3-fold polyethyleneimine (PEI, 1 μg/mL Polyscience), and then incubated at RT for 20 min. After incubation, the pre-made mixture was added to 293 T cells for 16 h and then replaced with growth medium. After incubation for 16 h, the media soup was collected and filtrated using a 0.45 μm PVDF membrane syringe filter (Millipore, Bedford, MA, USA). For transfection, gastric cancer cells (NCI-N87, MKN-28, AGS, MKN-1) were seeded in a 60 mm dish using 5 × 10^5^ cells. The next day, the cells were added mixture (2:1 ratio of virus soup and growth medium with 3 μg/mL polybrene (Sigma-Aldrich, St. Louis, Missouri, USA). After incubation for 12 h, the cells were replaced with growth medium 2 times. For selection, cells were treated with 3 μg/mL puromycin (Invitrogen, Cergy-Pontoise, France). The efficiency of the knock-out was tested using WB. For the colony formation assay, 1 × 10^3^ established cells were seeded in a 6-well plate. Approximately 2-weeks later, cells were washed using PBS and 500 μL Crystal Violet staining solution, including 0.05% Crystal Violet (Sigma-Aldrich), 1% formaldehyde, 1x PBS, and 1% MeOH, was added to each well for 20 min at RT. After incubation, cells were detected by imaging each well.

### Evaluation of IHC and CISH

Normal gastric foveolar epithelial cells served as an internal positive control for 15-PGDH and lymphocytes for COX-2 and PGE_2_ expression. For IHC scoring, we devised a semi-quantitative H-score ranging from 0 to 200, which was the sum of staining intensity [0 (no or weak staining), 1 (moderate), 2 (strong)] multiplied by the corresponding proportion in the tumor (0 to 100). CISH for miR-21 was scored with the highest intensity of staining: 0 (absent), 1 (weak), 2 (moderate), or 3 (strong).

### Statistical analysis

Statistical analysis was performed using PASW software (version 21; SPSS Inc., Chicago, IL). Differences between the two groups in terms of continuous variables were analyzed by using the Student t-test, paired t-test, and Mann–Whitney test. *P*-values < 0.05 were considered statistically significant.

## Electronic supplementary material

Supplementary article

## Data Availability

The authors declare that all the data in this manuscript are available.
