# Supplementary material for: MicroRNA-21 induces loss of 15-hydroxyprostaglandin dehydrogenase in early gastric tubular adenocarcinoma
Source: Sci Rep. 2018 Dec 7;8:17717. doi: 10.1038/s41598-018-36139-z (PMC6286338; doi:10.1038/s41598-018-36139-z)
Supplement: Supplementary file 1 — Supplementary article [file 41598_2018_36139_MOESM1_ESM.docx]

**Supplementary documents**

**MicroRNA-21 induces loss of 15-hydroxyprostaglandin dehydrogenase in early gastric tubular adenocarcinoma**

Young Soo Park*^1^, Jeong Hoon Lee*^2^, Deok-Beom Jung*^3^, Han-Byul Kim^3^, Jin-Hak Jung^3^, Sehyung Pak^3^, Yeon-Mi Ryu^3^, Hye Jin Park^3^, Yun-Yong Park^3^, Hwoon-Yong Jung^2^, and Seung-Jae Myung^2,3^

Department of ^1^Pathology and ^2^Gastroenterology, University of Ulsan College of Medicine, Asan Medical Center, Seoul, Korea; ^3^Biomedical Research Center, Asan Institute for Life Sciences

Corresponding author: Seung-Jae Myung, MD, PhD

Professor, Department of Gastroenterology, University of Ulsan College of Medicine, Asan Medical Center, Seoul, Korea

88 Olympic-Ro, 43-Gil, Songpa-gu, Seoul 05505, Korea

Phone: 82-2-3010- 3917

Fax: 82-2-476-0824

E-mail: sjmyung@amc.seoul.kr

Short running head: miR-21 in gastric tubular adenocarcinoma

*Young Soo Park, Jeong Hoon Lee and Deok-Beom Jung equally contributed to this study as co-first authors

**Supplementary Materials and Methods**

**Primary antibodies**

IHC staining was performed using following antibodies: anti-15-PGDH (1:5000), anti-COX-2 (1:400, #RB-9072, Thermo), and anti-PGE-2 (1:400, #ab2318, Abcam).

WB analysis was performed by the following antibodies: anti-beta-actin (1:10000, Sc-47778, Santa Cruz), anti-15-PGDH (1:1000, NB200-179, Novus Biologicals), anti-COX2 (1:1000, RB-9072-P0, Thermo), and anti-DNMT1 (1:1000, ab19905, Abcam).

**qRT-PCR primers**

The following primers were used for amplification:

15-PGDH

Forward 5′-TCTGTTCATCCAGTGCGATGT-3′

Reverse 5′- ATAATGATGCCGCCTTCACCT -3′

COX-2

Forward 5′- TGAAACCCACTCCAAACACA -3′

Reverse 5′- GAGAAGGCTTCCCAGCTTTT -3′

GAPDH

Forward 5′- ATGGGGAAGGTGAAGGTCG -3′

Reverse 5′- GGGGTCATTGATGGGAACAATA -3′

miR-21a

5′-UAGCUUAUCAGACUGAUGUUGA-3′ (miScript Primer Assay, Qiagen).

**MS-PCR**

Primer sequences for 15-PGDH MS-PCR were as follows:

Forward (U) 5′-GGGTATAAAAGTTGTGGTTGTGT-3′

Reverse (U) 5′-AAAAAAATTTCCACAACTAAACACCA-3′

Forward (M) 5′-GTATAAAGTCGCGGTCGCGC-3′

Reverse (M) 5′-AAATTTCCGCGACTAAACGCCG-3′

Methylation-specific PCR (MSP) was individually performed in 25 μL reaction volumes using the EpiTect MSP kit (Qiagen). The products were separated on a 2% agarose gel and detected by ethidium bromide staining. The PCR product of the methylated form was 189 bp and 195 bp for the un-methylated form. Methylation and unmethylated human control DNA was purchased from Qiagen (Hilden).

**Pyrosequencing**

Primer sequences for 15-PGDH pyrosequencing were as follows:

Forward: 5′-AYGTTTAGGGGGTAGGTGATATAG-3′

Biotinylated-Reverse: 5′-TTACCRTTCACRTACATAATACAA -3′

Sequencing primer: 5′-GTAGGGGGGTATAAAAG -3′

*NOTE: Y = C or T, R = A or G.

The PCR reaction was performed in a volume of 20 μL containing 20 ng converted gDNA, PCR premixture (Enzynomics, Seoul, Korea), 2 μL of 10 pmol/μL Primer-S, and 2 μL of 10 pmol/μL biotinylated-Primer-As. The amplification was carried out initiated at 95℃ for 10 min, followed by 45 cycles at 95℃ for 30 s, at 55℃ for 30 s, at 72℃ for 30 s, and a final extension at 72℃ for 5 min. The single-stranded DNA template was prepared from biotinylated PCR product using streptavidin Sepharose HP beads (Amersham Biosciences) as described by the PSQ 96 sample preparation guide. Sequencing was conducted on a PyroMark ID system using the PyroGold Reagents Kit (Biotage) according to the manufacturer’s instructions.

**CISH**

For CISH, 6 μm sections from each paraffin block were prepared. Sections were deparaffinized with xylene (3 × 5 min) and then rehydrated with ethanol solutions (99.9% - 96% - 70%) ending in PBS, pH 7.4. Proteinase-K (15 μg/mL, Exiqon, Vedbaek, Denmark) treatment was done in PK-buffer (5 mM Tris.HCl, pH 7.5, 1 mM EDTA, 1 mM NaCl, autoclaved) at 37°C for 20 min in a StatSpinHybridizer (Dako). After a PBS wash, the sections were dehydrated through an increasing gradient of ethanol solutions and air-dried. The LNA-probes were denatured by heating to 90°C for 4 min. Hybridization of the LNA-probe miR-21 (40 nM), scramble miR (40 nM), and U6 (1 nM) l was carried out in the automated Hybridizer at 50°C for 60 min. Stringent washes were performed in pre-heated SSC buffers, 1 × 5 min in 5× SSC and 2 × 5 min in 1× SSC and 0.2× SSC. Sections were blocked against unspecific binding in blocking solution for 15 min at room temperature (RT). Alkaline phosphatase (AP)-conjugated anti-DIG (Roche, Mannheim, Germany) 1:400 was incubated for 60 min at RT for immunologic detection. After a PBS-T wash, the substrate enzymatic reaction was carried out with NBT/BCIP (Roche) at 30°C in the hybridizer for 120 min. The reaction was stopped with a 2 × 5 min wash in KTBT buffer (50 mM Tris-Hcl, 150 mM NaCl, 10 mM KCl). Counter stain with nuclear fast red (Vector Laboratories, Burlingame, CA) was done at RT for 1 min and then rinsed in tap water, dehydrated through an increasing gradient of ethanol solutions, and mounted with Eukitt mounting medium (VWR, Herlev, Denmark).

**Supplementary Figure 1. Expression of 15-PGDH, COX-2 and PGE2 in well differentiated adenocarcinoma.**


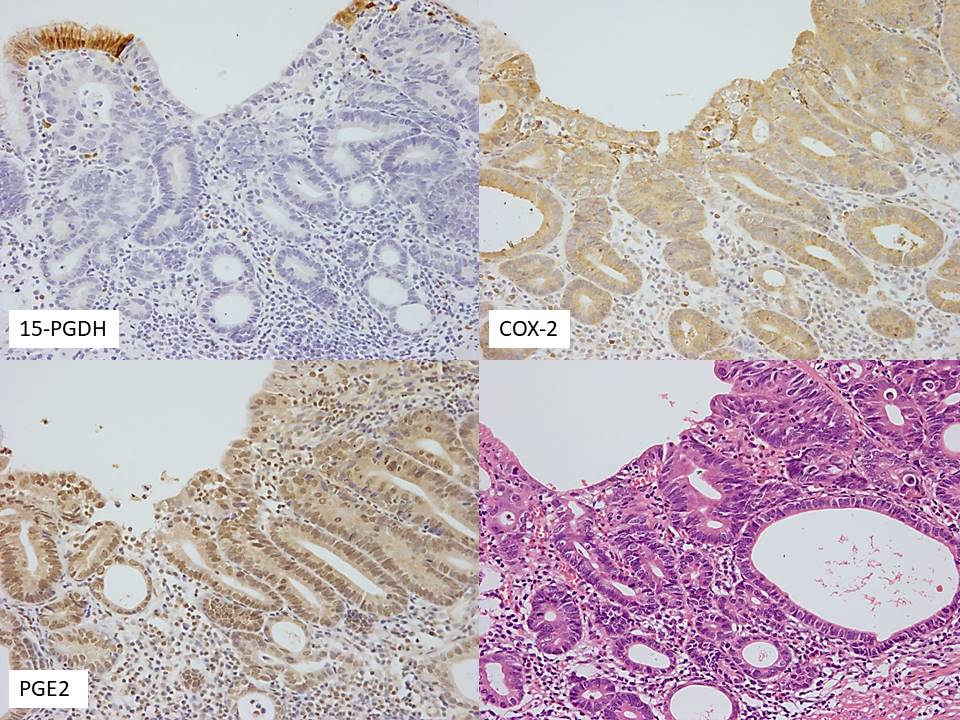


Photomicrographs of a representative case of very well differentiated adenocarcinoma. 15-PGHD expression is lost in the carcinoma area (dysplastic cells) whereas COX-2 and PGE2 are clearly positive (original magnification, x200).

**Supplementary Figure 2. De-methylation study with 5-aza-dC treatment for 72 h in gastric cancer cell lines**.


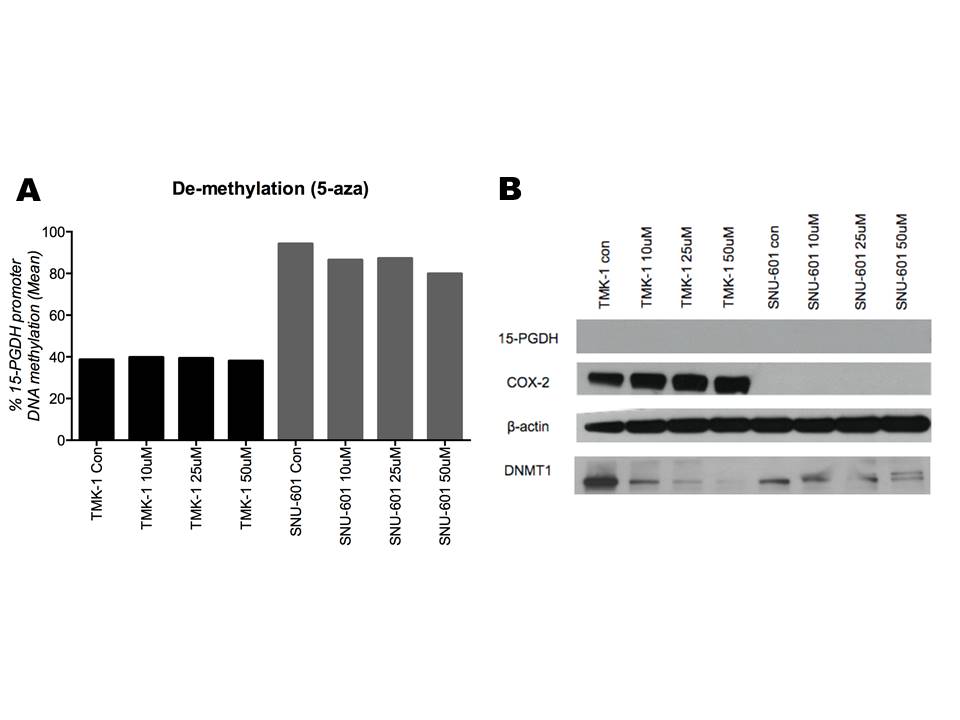

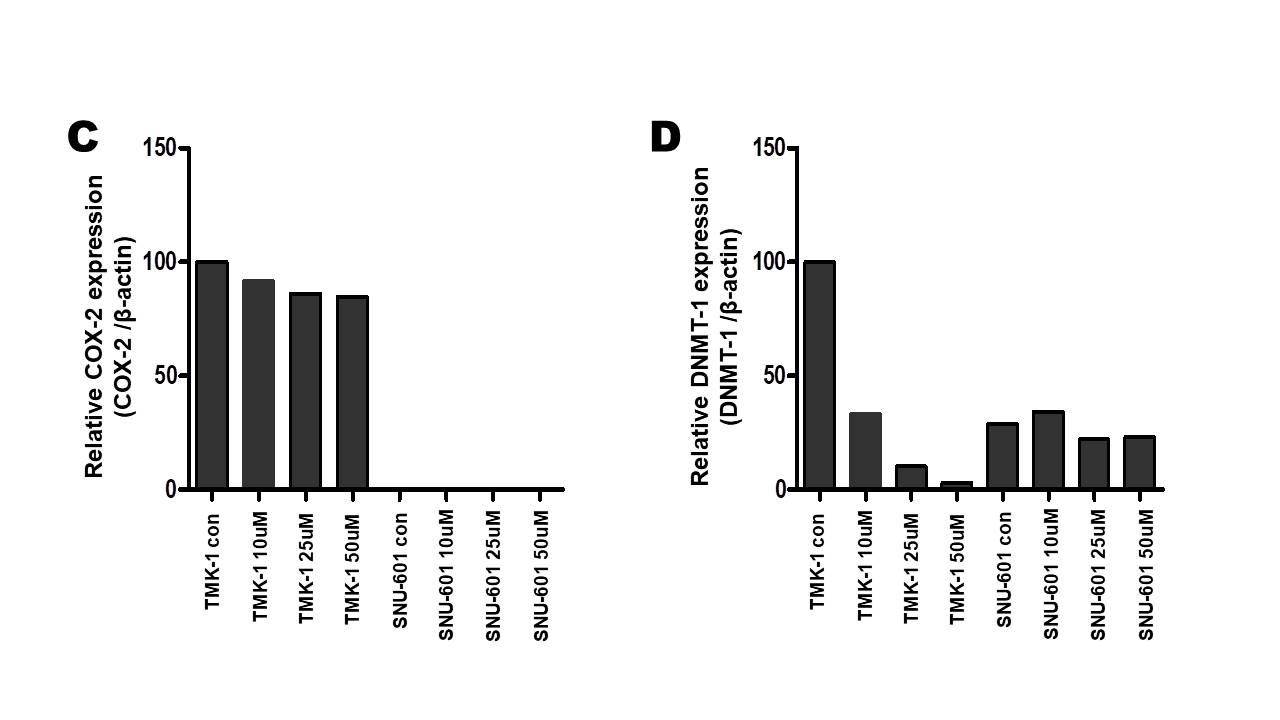


Pyrosequencing showed no change in 15-PGDH promoter methylation (**A**) and 15-PGDH protein expression was not recovered (**B**). Normalized western blot band densities using the actin density in each column confirms no change of COX-2 (C) expression. The expression of DNMT1 was diminished after 5-aza-dC treatment in TMK-1 but not in SNU-601.

**Supplementary Figure 3. Overexpression of 15-PGDH inhibits cell growth.**


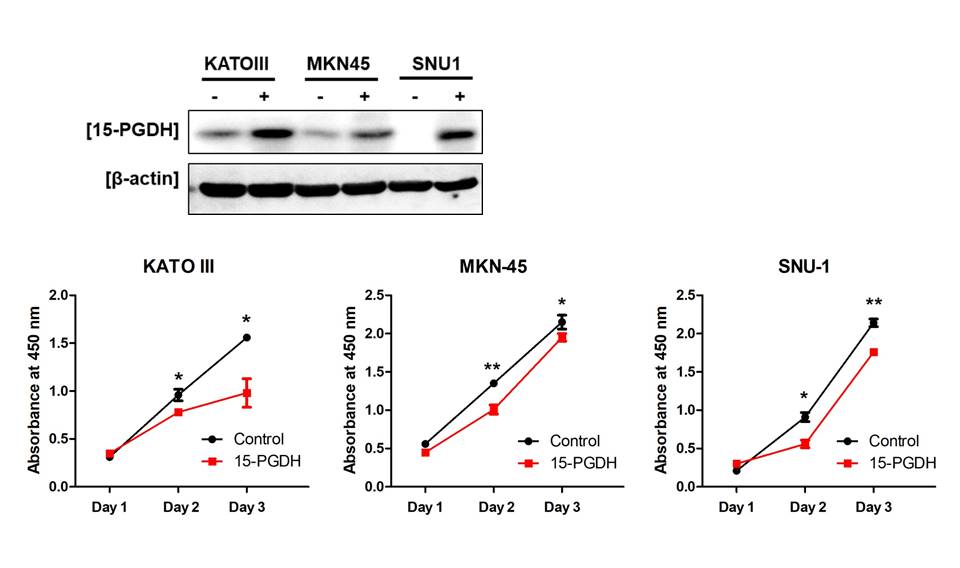


The western blots (upper panel) show increased protein level in transfected cells. The growth curves (lower panel) show inhibited cell growth after 15-PGDH overexpression (red) in KATOIII, MKN-45 and SNNU-1 cells (* P < 0.05, ** P < 0.001)

**Supplementary Figure 4. miR-21 transfected cells make more number of colonies.**


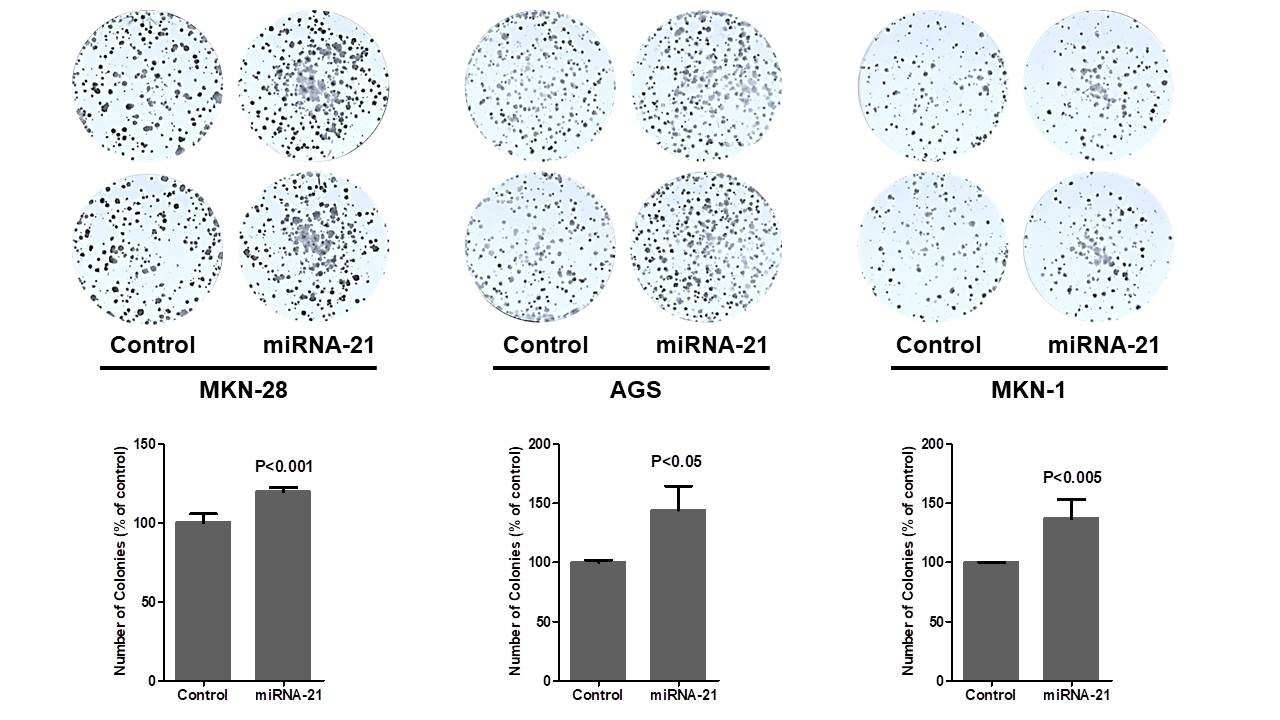


Colony formation assays comparing baseline and miR-21 transfected cells (MKN-28, AGS, MKN-1) show increased number of colonies after miR-21 transfection.

**Supplementary Figure 5. miR-21 CISH in retrospectively collected ESD cases.**

**
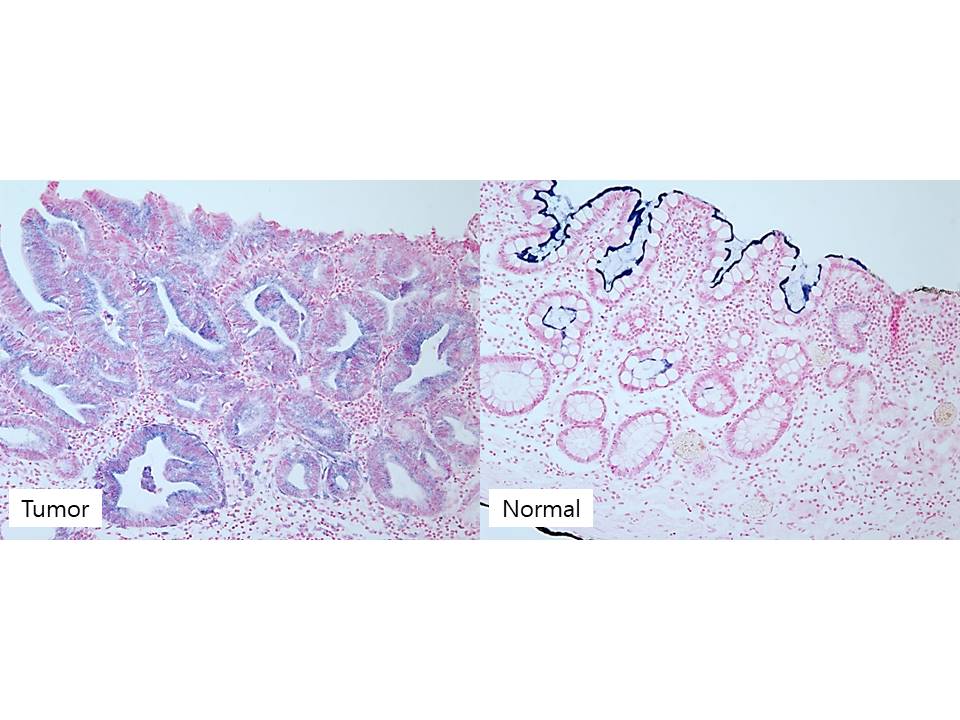
**

In this representative photomicrographs, miR-21 CISH staining is 3+ in the tumor area and 0 in the adjacent normal area (original magnifications, x200).

**Supplementary Figure 6. Impact of *H. pylori* infection on miR-21 expression.**


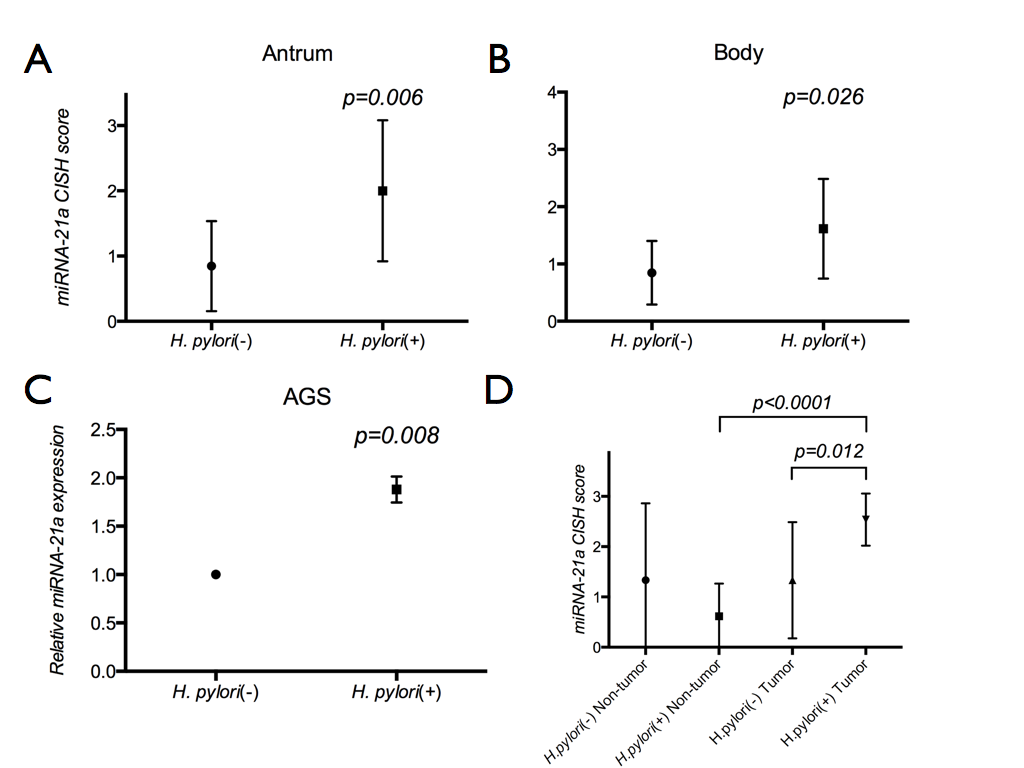


**A and B**, comparison of miR-21 CISH scores from 14 normal subjects according to *H. pylori* infection status. miR-21 was higher in *H. pylori*-infected subjects irrespective of the site of the biopsy. **C**, in AGS cell line, miR-21a expression level increased after *H. pylori* infection was detected by qRT-PCR. **D**, scores of miR-21 CISH in the 16 ESD specimens according to *H. pylori* infection (13 positive and 3 negative) status and non-tumor vs. tumor. miR-21 CISH score was higher in the tumor samples than in the normal samples in *H. pylori*-infected patients. In addition, miR-21 was higher in the tumor samples of *H. pylori*-infected patients compared to that of uninfected patients.

**Supplementary Figure 7. 15-PGDH, COX-2, and PGE2 IHC in early signet ring cell carcinoma**


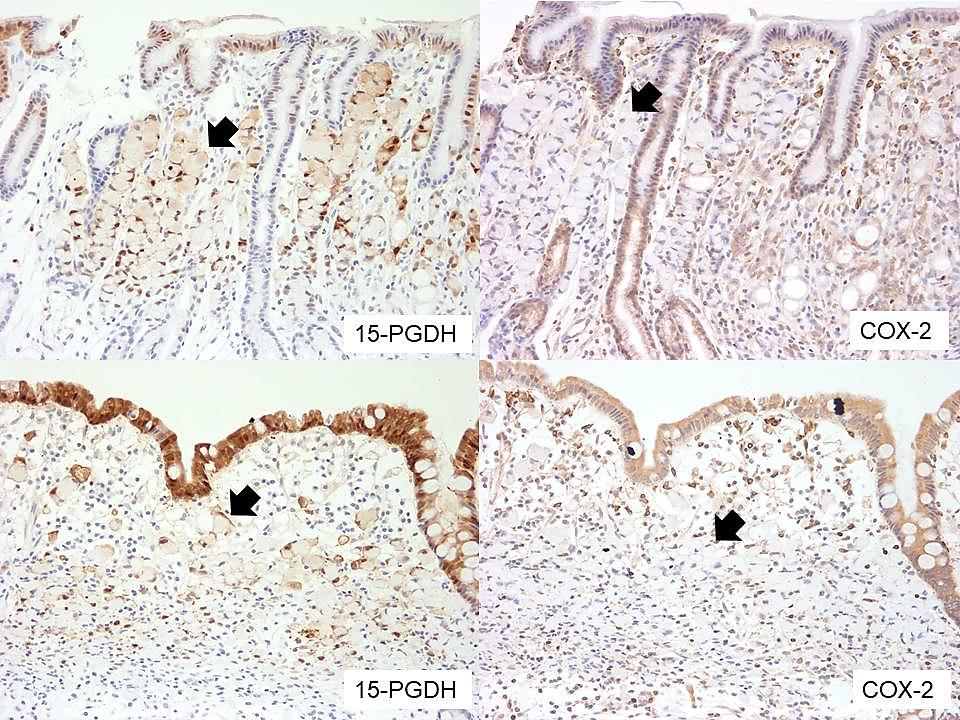


Representative photomicrographs of IHC staining for 15-PGDH (left column) and COX-2 (right column) in early gastric signet ring cell carcinomas. The tumor cells (arrows) are clearly positive for 15-PGDH (internal positive control: overlying foveolar epithelial cells), but are negative for COX-2 (internal positive control: lymphocytes. Original magnifications, x200).

Supplementary Table 1. Characteristics of the gastric cancer cell lines

| Cell line | 15-PGDH | | | COX-2 | |
| --- | --- | --- | --- | --- | --- |
|  | relative protein | relative mRNA  (AGS as standard score) | MS PCR  (pyrosequencing %) | relative protein | mRNA  (AGS as standard score) |
| TMK-1 | negative | 0 | methylated (40%) | ++++ | 2000 |
| AGS | ++++ | 100 | un-methylated (0%) | negative | 100 |
| SNU-668 | negative | 0 | un-methylated (0%) | ++ | 400 |
| MKN-45 | + | 94 | un-methylated (0%) | + | 388 |
| MKN-28 | +++++ | 434 | un-methylated (0%) | negative | 12 |
| SNU-484 | negative | 7 | un-methylated (0%) | negative | 30 |
| SNU-216 | negative | 0 | methylated (14%) | negative | 2 |
| SNU-5 | negative | 13 | un-methylated (0%) | negative | 4 |
| SNU-601 | negative | 0 | methylated (95%) | negative | 1 |
| SNU-719 | negative | 21 | un-methylated (0%) | negative | 9 |
| SNU-1 | negative | 0 | methylated (99%) | +++ | 177 |
| SNU-638 | negative | 5 | un-methylated (0%) | negative | 1 |
| KATO III | ++ | 61 | un-methylated (0%) | negative | 12 |
| MKN-1 | ++++ | 170 | un-methylated (0%) | negative | 49 |
| NCI-N87 | +++ | 110 | un-methylated (0%) | negative | 15 |
